# Hepatitis E in pigs in Israel: seroprevalence, molecular characterisation and potential impact on humans

**DOI:** 10.2807/1560-7917.ES.2018.23.49.1800067

**Published:** 2018-12-06

**Authors:** Rachel Shirazi, Paolo Pozzi, Marina Wax, Itay Bar-Or, Efrat Asulin, Yaniv Lustig, Ella Mendelson, Ziv Ben-Ari, Eli Schwartz, Orna Mor

**Affiliations:** 1 Central Virology Laboratory, Ministry of Health, Tel-Hashomer, Ramat-Gan, Israel; 2 These authors contributed equally to this article; 3 Israel Ministry of Agriculture and Rural Development Plant Protection and Inspection Services, Veterinary Services Beit Dagan, Beit Dagan, Israel; 4 School of Public Health, Sackler Faculty of Medicine Tel Aviv University, Tel Aviv, Israel; 5 Liver Diseases Center, Chaim Sheba Medical Center, Tel Hashomer, Ramat-Gan, Israel; 6 Sackler Faculty of Medicine, Tel Aviv University, Tel Aviv, Israel; 7 Center for Geographic Medicine and Tropical Diseases, Chaim Sheba Medical Center, Tel Hashomer, Ramat-Gan, Israel

**Keywords:** Hepatitis E, HEV-G3, sewage, HEV RNA, pigs, Seroprevalence

## Abstract

**Introduction:**

The zoonotic hepatitis E virus (HEV) genotype 3 (HEV-G3) has become a common cause of acute and chronic hepatitis among humans worldwide. In Israel, while HEV-3 sequences have previously been detected in sewage, only the non-zoonotic HEV-G1 genotype has been found in samples from human patients.

**Aim:**

In this pilot study, we aimed to assess the status of HEV in a sample of the swine population and among swine farm workers in Israel.

**Methods:**

Pig blood (n = 141) and faecal samples (n = 39), pig farm sewage samples (n = 8) and blood from farm workers (n = 24) were collected between February 2016 and October 2017. Anti-HEV IgG was detected using the Wantai assay. HEV RNA was analysed with the RealStar HEV kit. HEV open reading frame 1 fragments amplified from representative HEV RNA-positive samples were used for phylogenetic analysis.

**Results:**

Overall prevalence of HEV antibodies in pigs was 75.9% (107/141). HEV RNA was detected in plasma (2.1%, 3/141), faecal (22.8%, 18/79) and pig sewage (4/8) samples. Pig and sewage-derived viral sequences clustered with previously identified human sewage HEV-G3 sequences. Most pig farms workers (23 of 24) were HEV-seropositive; none was viraemic or reported previous clinical signs.

**Conclusions:**

This study showed that domestic pigs in Israel are infected with HEV-G3. The high HEV seropositivity of the farm workers together with the previous identification of this virus in human sewage suggests circulation to humans. The clinical impact of these findings on public health should be further explored.

## Introduction

Hepatitis E virus (HEV), which is primarily transmitted via the faecal-oral route, is a major causative agent of acute viral hepatitis in developing countries. At least 20 million HEV infections occur annually, and while most infections result in a self-limiting disease, ca 60,000 fatalities are reported every year [1]. Moreover, up to 30% mortality in women in the third trimester of pregnancy has been recorded [2]. In recent years, reports on the involvement of HEV viral hepatitis in Europe and non-European Mediterranean countries have been accumulating. Both acute and chronic viral hepatitis cases were identified. Two of the eight known HEV genotypes [3] are most frequently identified in these regions: HEV-G1, which is considered endemic in Asia, Africa and South America and infects humans through contaminated water [4], and HEV-G3, which is a zoonotic virus infecting pigs and other animals and is primarily transmitted to humans through the consumption of infected meat [5]. Both genotypes were detected in clinical and environmental samples, however, while HEV-G1 infection results only in acute viral hepatitis, infection with different HEV-G3 subtypes could also result in persistent chronic hepatitis [4] and is considered to be the main cause of HEV-related chronic viral hepatitis in Europe. Thus, to reduce the risk for blood-borne transmission of HEV-G3 sequences from donors chronically infected with this virus, several European countries have already initiated HEV screening of blood donations [6].

The incidence of HEV infection in Israel is very low; only two to three cases per year have been reported between 1997 and 2012 [7]. Most cases were travellers returning from HEV-endemic countries who presented with acute viral hepatitis. When assessed, HEV-G1 was the only HEV genotype identified. Assuming that viral circulation can be revealed by environmental sampling, we have previously shown that a subset of sewage samples collected between 2014 and 2015 was HEV-positive [8]. Surprisingly, sequence analysis revealed HEV-G3 sequences (of subtype G3f) in those RNA-positive samples, although this genotype has never been identified in any of the clinical cases in Israel.

A possible source for HEV-G3 sequences are domestic pigs. Although pig consumption is not common in Israel, 90,000 pigs are being farmed at any given time on 24 swine breeding farms. Of these, 80,000 are bred on 23 farms in the northern region (West Galilee) and 10,000 are bred on a single farm in the southern region of Israel (Negev). The population of sows (breeders) in Israel is around 14,000. About 200,000 pigs are slaughtered per year, at the age of 6 months, exclusively for local consumption, yet, HEV exposure of humans has never been documented. HEV infection in pigs is asymptomatic and short. It can be detected in the blood for a short period of several weeks after infection. Virus shedding via the pig faeces is longer and can be observed for up to 155 days [9].

In this pilot study, we aimed to assess the status of HEV in a sample of the swine population and among swine farm workers in Israel.

## Methods

### Samples

Blood samples from 141 randomly selected pigs of different age groups were collected between February 2016 and October 2017. The pigs were from the southern farm (n = 38) and from three northern farms (n = 103). These three farms were selected to ensure a geographical distribution that is representative of the location of all 23 northern farms [10]. Approximately 50 g of fresh faeces (n = 39) were collected from the floor of the pig pens, each holding pigs of similar age (1.5–6 months of age; different pens by age group). Eight raw sewage samples, 500 mL each, were also collected; six were collected between 2016 and 2017, during three different visits, from the single sewage pipeline serving all northern pig farms, and two were collected in 2016, during two different visits to the southern farm.

Blood samples from all 24 workers in two of the northern farms (including two veterinarians) were also assessed. All samples were transported on ice to the laboratory and processed within 24 hours.

### Ethical approval

The study was approved by the Ethical Committee of the Sheba Medical Center (approval number: 4255–17-SMC) and written informed consent was obtained from the participants.

### Laboratory and data analysis

Anti-HEV IgG was detected in sera using the commercial enzyme-linked immunosorbent assay (ELISA) total IgG (for pig sera) or IgG (for human sera) HEV assays (Wantai Biopharmaceutical, Inc. Beijing, China). Serum specimens exhibiting an absorbance value greater than the cut-off value, calculated as the mean absorbance value for negative controls + 0.16, were considered positive for anti-HEV antibodies.

Total nucleic acids were extracted from 0.5 mL plasma (human and pigs), 1 mL concentrated sewage samples and 0.2 mL faeces suspension, using the NucliSENS EasyMag (bioMérieux SA, Marcy l’Etoile, France), as previously described [8,11]. HEV RNA was analysed with the RealStar HEV RT-PCR kit, version 2.0 (Altona Diagnostics GmbH, Hamburg, Germany). HEV open reading frame 1 (ORF1) fragments were amplified from representative HEV RNA-positive samples and sequenced as previously described [8]. Sequences (GenBank accession numbers: MH253054–MH253056) were assembled into a phylogenetic tree and compared with human sequences identified during this study period (MH253049-MH253053) and human and human-sewage HEV sequences previously identified in Israel [8] and with 10 reference sequences [12] retrieved from GenBank, using MEGA 6.0 [13].

## Results

### Hepatitis E virus in pigs and in sewage from pig farms

**Figure 1:**
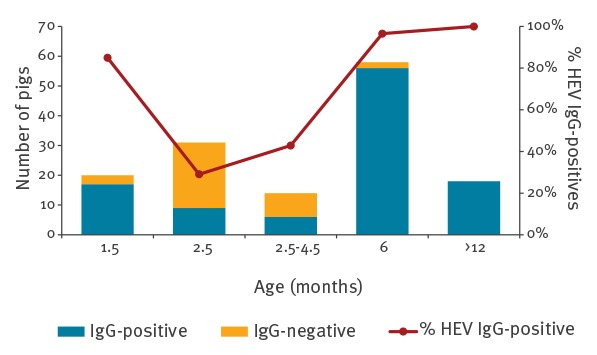
Number and prevalence of pigs with IgG antibodies against hepatitis E virus, by age group, Israel, February 2016–October 2017 (n = 141)

Anti-HEV antibodies were identified in 75.9% (107/141) of the plasma samples from pigs, with seroprevalence differing in the various age groups (Figure 1, Table). Seventeen of 20 samples from 1.5-month-old pigs, 16 of 45 samples from 2.5–4.5-month-old pigs and 74 of 76 samples from pigs aged 6 months or older (sows) were anti-HEV-positive. In addition, 2.1% (3/141) of the blood samples were HEV RNA-positive, all collected from IgG-negative 2.5–4.5 month-old pigs. Plasma samples from pigs aged 6 months or older (n = 76) were all HEV RNA-negative.

Anti-HEV IgG was detected using total IgG ELISA assay (Wantai Biopharmaceutical, Inc. Beijing, China) in sera from 141 randomly selected pigs collected between February 2016 and October 2017.

**Table:** Anti-hepatitis E virus IgG and hepatitis E virus RNA-positive samples in blood, faeces and sewage from pigs in northern and southern breeding farms, Israel, February 2016–October 2017

Region of breeding farms	Age (months)	Blood (n = 141)			Faeces (n = 79)		Sewage (n = 8)	
		All samples tested	IgG-positive samples	RNA-positive samples	All samples tested	RNA-positive samples	All samples tested	RNA-positive samples
North Israel	1.5	20	17	0	15	0	6	2
	2.5	21	7	2	16	9		
	3.5	14	6	1	24	6		
	4.5				24	3		
	6	30	28	0	NA	NA		
	>12	18	18	0	NA	NA		
South Israel	2.5	10	2	0	NA	NA	2	2
	6	28	28	0	NA	NA		

NA: not available.

HEV RNA was detected in 22.8% (18/79) of the faecal samples, all positive samples being from pigs 2.5–4.5 months of age. The frequency of seropositivity and HEV RNA positivity was similar across the tested breeding farms (data not shown). HEV RNA was also detected in half (4/8) of the sewage samples collected from the swine sewage line.

**Figure 2:**
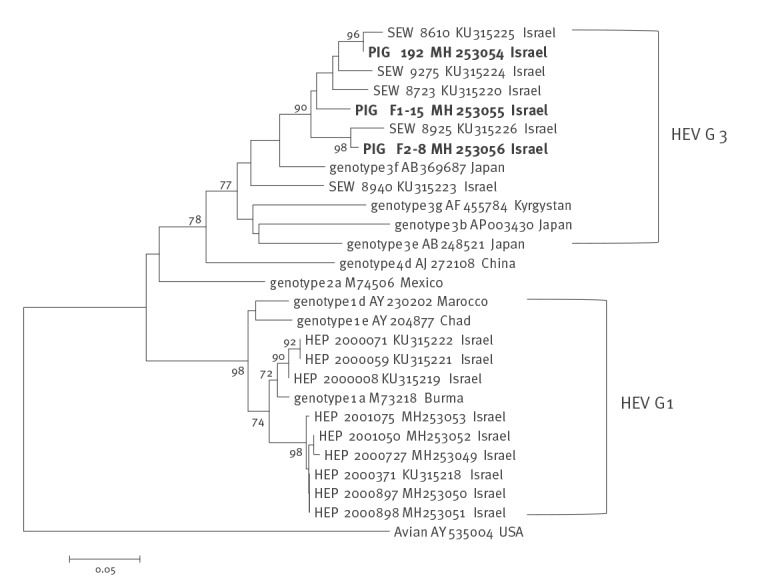
Phylogenetic analysis of the Israeli isolates of hepatitis E virus, Israel, February 2016–October 2017 (n = 8)

Sequencing of a 304 bp fragment from ORF1 from three representative HEV-RNA positive faecal and blood samples identified HEV-G3 (specifically similar to the G3f subtype) sequences which clustered with HEV sequences identified previously (in 2015) in sewage samples collected from human urban sewage facilities (Figure 2).

HEV: hepatitis E virus.

A phylogenetic tree of three pig- and five human-derived HEV isolates identified in this study, nine isolates identified in Israel in previous studies [8] and 10 prototype sequences obtained from GenBank (with Avian HEV as out-group) was constructed by Clustal W pairwise alignment of the 304 bp fragment (51–354 bp in AB69687) from the HEV ORF1.The prototype sequences are identified by the GenBank accession number and country of origin. The sequences identified in Israel are defined by their origin (pig, HEP = human, SEW=human sewage), sample numbers and accession numbers (KU315218–KU315226, MH253049-MH253056). Bootstrap values are indicated for the major nodes as data obtained from 1,000 replicates.

### HEV in farm workers

HEV seroprevalence was assessed in 24 individuals working in three of the northern pig farms. They were all men, with a median age of 43 years (range: 17–66 years) and a median period of exposure to pigs of 15 years (range: 0.3 months–53 years). Most (20/24) were Christians (18 Arabs and two non-Arab Catholics), three were foreign workers (Thai Buddhists) and one was Jewish (one of the veterinarians). All non-Jewish workers reported consuming pork. Nearly all (23/24), including the veterinarian Jew (who does not eat pork), were HEV IgG-positive. A Thai foreign worker who had been working at the farm for only 3 months, was the only seronegative individual. All 24 were negative for HEV RNA in plasma. None recalled any previous clinical signs of viral hepatitis.

## Discussion

In this study, we have demonstrated that HEV is present on domestic pig farms. In our pilot investigation, three quarters of all pigs were anti-HEV seropositive. This rate is similar to the reported rate of HEV in pigs in several European countries [14-16] and confirms that HEV is circulating among farmed pigs in Israel. The highest seroprevalence was observed in young pigs, 1.5 months old and in pigs aged 6 months and older. The peak identified early in life probably reflects the presence of maternal antibodies, which gradually declines after birth. Others have also shown that on HEV-positive farms, newborn pigs become susceptible to HEV infection between weeks 7 and 9, when serum levels of the maternal antibodies decline, and that most (81%) of the pigs aged 20–30 weeks are already anti-HEV IgG-positive [17,18].

Similar to other reports [19], we identified HEV RNA-positive blood and faecal samples in 2.5–4.5 month-old pigs. HEV RNA was mainly identified in faecal and not in blood samples, probably because blood viraemia lasts for a shorter period of time than faecal HEV shedding [20,21]. The high prevalence of anti-HEV antibodies identified here and the prevalence of HEV RNA in samples from 2.5–4.5 months-old pigs but not in samples from pigs aged 6 months and older suggests that pigs at the age of slaughter (6 months in Israel) are HEV RNA-negative and therefore cannot infect humans. This conclusion, however, should be thoroughly explored in a larger number of pigs, as conflicting results have been reported: While a study in Spain showed that animals that were in contact with the virus throughout their life span were seronegative at slaughtering [22], pigs in France exposed to the virus throughout their lifetime were found to be positive for HEV RNA in bile, liver and/or faeces at the time of slaughter [23]. Collectively, these results indicate that swine may remain susceptible to HEV infection at any age, even at slaughter.

Our local HEV-G3 sequences were most similar to the HEV-G3f and HEV-G3e subtypes identified in Japan [24] and to HEV-G3 strains reported in France [12,25]. This may suggest transmission between countries through international trading as already demonstrated when HEV sequences in pigs raised in Nigeria showed nucleotide identity to Japanese and European HEV strains [26,27]. The majority of the pigs raised in Israel belong to mixed breeds (Large White X Landrace, Duroc and Pietrain) and, while there is no import of live pigs to Israel, semen vials for artificial insemination are regularly imported from different European countries. Although hypothetically possible, transmission of HEV from semen has never been reported. Therefore, it is currently impossible to trace back the HEV sequences we identified here, and further studies are needed to explore this issue. The similarity observed between the HEV sequences from pigs identified here to those previously identified in sewage treatment facilities located in Haifa, Tel Aviv and Beer Sheva [8] suggests that circulation of HEV-G3 between pigs and humans is ongoing.

HEV IgG seropositivity in almost all farm workers suggests that they had all been exposed to the virus. As no clinical signs of viral hepatitis were recalled, we assume that HEV infection in these individuals was silent. Previously, we have reported a much lower overall seroprevalence (10.6%) for anti-HEV antibodies in the general Israeli population [28]. Higher prevalence of antibodies to HEV virus in swine workers compared with normal blood donors has already been reported in the United States and other countries [29,30] and demonstrates the high risk for HEV infection among individuals regularly exposed to infected animals.

Cooking the meat thoroughly is the most efficient method to inactivate HEV and to prevent food-borne HEV infection in humans [31]. However, even in countries where meat products are well-cooked and served in the form of stew or are preserved in salt, food-borne transmission of HEV occurs [26]. Both Judaism and Islam prohibit eating pork and pork products. Moreover, Israel has previously legislated the Meat Law that prohibits all imports of non-kosher meats [32]. However, a market for pork definitely exists in Israel and is based solely on the local production. Israeli Christian Arabs may consume it, as may foreign workers from the Far East (Thailand, China or the Philippines) and a minority of the Jewish population. As almost all HEV-seropositive farm workers reported pork consumption, under-cooked meat as a source for HEV infection for this population cannot be ruled out.

Our study has several limitations. We only sampled farm workers from three of the farms, suggesting a potential selection bias. The farm management and biosecurity practices were not assessed. Our recruitment and ultimately our sample size for swine blood and faecal samples was limited and small. The size of the HEV fragment used for phylogenetic analysis was small possibly affecting the quality of the HEV-G3 subtyping. Nevertheless, this is the first report that identified a high rate of HEV infection among pigs of different ages on all swine farms tested in the country. As most of the pigs in Israel are held in high-density breeding areas where HEV could be easily transmitted [33], and as the swine market is only local, circulation of HEV on all other Israeli farms can be expected. The close similarity between the HEV strains found previously in urban sewage and those we found on pig farms is alarming as no direct connection between these facilities exists. These results indicate that adequate safety actions should be taken to prevent HEV infection when handling pigs and when consuming local pig products. A national sampling frame for pigs covering all farms and all ages, especially before slaughter, and for biosecurity practices on the local farms, is warranted. Longitudinal studies enabling the analysis of a larger number of HEV RNA-positive samples could be beneficial for our understanding of the age of infection and the viral kinetics and will allow further investigation of the molecular epidemiology of HEV in Israel. Risk factors for people who work with pigs should be explored and correlated with HEV IgG-positivity.

A recently published survey of acute HEV in Israel revealed that between 1993 and 2013, 68 cases were reported as having HEV infection of whom 40% were not travel-related [34]. Unfortunately, the genotype of the autochthonous infections could not be elucidated. Our results may suggest that exposure to HEV-G3 could have been responsible for some of these cases. A better understanding of the consequences of infection with the local HEV-G3 sequences in naïve individuals is warranted.
